# Pulsed Electromagnetic Fields Increase Pigmentation through the p-ERK/p-p38 Pathway in Zebrafish (*Danio rerio*)

**DOI:** 10.3390/ijms19103211

**Published:** 2018-10-17

**Authors:** Yu-Mi Kim, Han-Moi Lim, Hyang-Seon Ro, Ga-Eun Ki, Young-Kwon Seo

**Affiliations:** 1 Department of Medical Biotechnology (BK21 Plus Team), Dongguk University, Goyang-si 10326, Korea; kjmtik@nate.com (Y.-M.K.); gksahl321@gmail.com (H.-M.L.); gigaeun1104@gmail.com (G.-E.K.); 2 R&D Center, NOWCOS, Seoul 08506, Korea; rohs@nowcos.co.kr

**Keywords:** pigmentation, melanogenesis, electromagnetic field, zebrafish, *Danio rerio*, vitiligo

## Abstract

Melanogenesis is a biological process resulting in the production of melanin pigment, which plays an important role in the prevention of sun-induced skin injury, and determines hair and skin color. So, a wide variety of approaches have been proposed to increase the synthesis of melanin. This study evaluated the effects of pulsed electromagnetic fields (PEMFs) on the pigmentation of zebrafish (*Danio rerio*) in vivo. We stimulated pigmentation in zebrafish by using specific frequencies and intensities of PEMFs. This study focuses on pigmentation using PEMFs, and finds that PEMFs, at an optimal intensity and frequency, upregulate pigmentation by the stimulated expression of tyrosinase-related protein 1 (TRP1), dopachrome tautomerase (DCT) through extracellular signal-regulated kinase(ERK) phosphorylation, and p38 phosphorylation signaling pathways in zebrafish. These results suggest that PEMFs, at an optimal intensity and frequency, are a useful tool in treating gray hair, with reduced melanin synthesis in the hair shaft or hypopigmentation-related skin disorders.

## 1. Introduction

Melanin is a unique pigmented biopolymer synthesized by specialized cells known as melanocytes, which are dendritic cells that exist in relatively minor populations in the skin, hair, eyes, and other locations [1]. The natural tanning process occurs as a response to exposure to UV radiation. [2]. Several recent studies have focused on the treatment of diseases caused as a result of hypopigmentation of the skin or hair. Vitiligo is a common depigmenting skin disorder, with a worldwide prevalence of 0.5–1% [3]. A variety of physical treatments are used to induce melanin production in the melanocytes of vitiligo patients, who are characterized by a partial loss of melanocytes from the epidermis of the skin. Recently, a lot of alternative therapies have been proposed as vitiligo treatments. Narrow-band UVB radiation has been shown to be effective in the treatment of vitiligo [4]. In addition, a variety of other approaches for inducing pigmentation have been proposed, including the use of an excimer laser (308 nm) [5,6], monochromatic emission at 355 nm (UVA1) laser [7], helium–neon laser [8], neodymium-doped yttrium aluminum garnet(Nd: YAG) laser [9], and Q-switched ruby laser [10,11]. Safe methods to promote melanogenesis would be valuable in medicine and cosmetics. 

The use of pulsed electromagnetic fields (PEMFs) has recently been explored as an effective method because of its non-invasiveness, safety, lack of side-effects, convenience, and superior treatment prospects in the treatment of several refractory bone diseases, such as non-unions and the delayed healing of fractures [12]. Furthermore, several studies have suggested that electromagnetic fields (EMFs) are effective in cartilage repair [13], in reducing diabetic neuropathic pain [14], in adult hippocampal neurogenesis [15], in angiogenesis [16], and in skin wound repair [17]. Our previous studies have shown that PEMF induces melanogenesis in B16F10 melanoma and human melanocyte cells [18,19,20]. Pigmentation is a complex process involving many factors. Microphthalmia-associated transcription factor (MITF) is the master gene involved in pigmentation, and it controls several crucial mechanisms in melanocytes, such as melanogenesis, dendricity, and proliferation, in response to environmental factors [21]. The activity of melanogenic enzymes and melanosome transfer can affect pigmentation. Tyrosinase, tyrosinase-related protein 1 (TRP 1), and TRP 2 are the melanocyte-specific enzymes involved in melanin biosynthesis [22]. Recently, it was reported that melanogenesis is also controlled by various intracellular signaling molecules, such as mitogen-activated protein kinases (MAPK) [23].

The zebrafish (*Danio rerio*) has become a favored model for biochemical studies because it is an efficient and robust alternative to conventional animal experiments [24]. It is a small tropical freshwater fish, and is a useful vertebrate model organism because of its small size, large clutches, transparent body, and physiological similarity to mammals, in addition to its low cost of breeding [25]. In addition, zebrafish have melanin pigmentation on their body surface and rapidly alter their pigmentation in response to environmental changes, which enables a facile investigation of pigmentation without the need for complicated experimental procedures [26].Therefore, the zebrafish is an ideal model for the study of melanogenesis in the context of pigmentation. In the present study, we applied PEMFs for the stimulation of pigmentation in zebrafish. Zebrafish larvae were stimulated at intensities of 2, 4, and 20 G, at a constant frequency of 60 Hz. In order to verify the changes in pigmentation, we performed melanin assays, a Western blot analysis, and immunohistochemical staining. Specifically, we analyzed the changes in ERK and p38 signaling associated with MITF regulation, due to the PEMFs. 

## 2. Results

### 2.1. Melanin Assay

**Figure 1:**
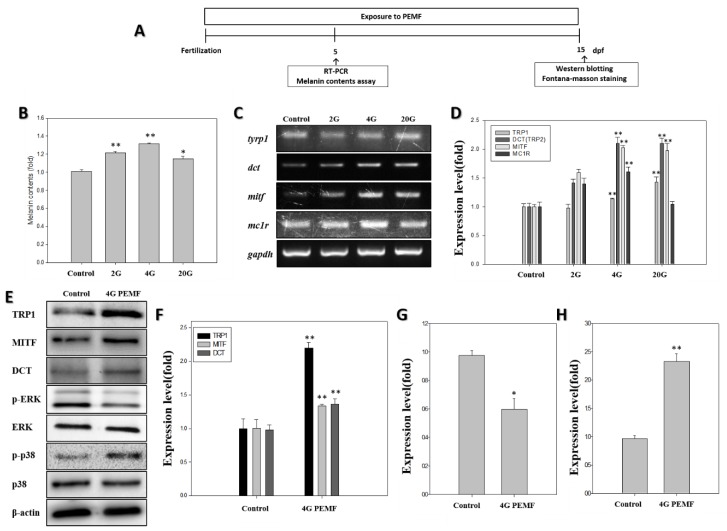
Effect of pulsed electromagnetic fields (PEMFs) on the pigmentation of zebrafish after exposure to PEMFs. (**A**) Schematic representation of the schedule of the zebrafish pigmentation study. After PEMF exposure, the melanin content was determined. (**B**) The melanin content in zebrafish (n = 80) was detected using the melanin content assay at 5 dpf. (**C**) The mRNA expression, detected using reverse transcription polymerase chain reaction, results in zebrafish (n = 20) after exposure to PEMFs at 5 dpf. (**D**) The mRNA expression of melanogenesis-related genes, using glyceraldehyde-3-phosphate dehydrogenase (GAPDH) as the reference gene. The effect of PEMF on the protein levels of tyrosinase-related protein-1 (TRP1), microphthalmia-associated transcription factor (MITF), dopachrome tautomerase (DCT), extracellular signal-regulated kinase (ERK), p-ERK, p-p38, p38, and b-actin in zebrafish (n = 30) at 15 dpf. (**E**) The Western blotting analysis of the pigmentation-related proteins; (**F**) TRP1, MITF, and DCT expression level; (**G**) p-ERK expression; (**H**) p-p38 expression. Each bar represents the mean ± standard error of the independent experiments performed in triplicate (n = 5). * *p* < 0.05, ** *p* < 0.01, compared to the control.

To test whether the PEMFs induced pigmentation in zebrafish, the amount of melanin was determined using a melanin content assay after exposure to PEMFs. The melanin content of the PEMF-treated zebrafish larvae was more than that of the untreated zebrafish larvae (Figure 1B). The 2 G group showed an increase of 1.22-fold, the 4 G group of ~1.32-fold, and the 20 G group showed an approximately 1.16-fold increase over the negative control group. The larvae exposed to 4 G exhibited a particularly large increase relative to the controls. These results suggest that the PEMFs, especially at 4 G, increased pigmentation in zebrafish.

### 2.2. RT-PCR

The mRNA expression levels of the key melanogenesis-related genes, *dct*, *tyrp1*, *mitfa*, and *mc1r*, were measured at 5 dpf (days post-fertilization), as shown in Figure 1. The *Dct* mRNA levels in the zebrafish exposed to PEMFs were on average 1.4-fold higher than those in the control. The zebrafish exposed to PEMFs at 4 G showed a *dct* expression ~2.0 times higher than that in the controls. The *mitfa* expression levels increased at all intensities of the PEMFs. The *mitfa* mRNA levels in the zebrafish exposed to 4 G PEMFs were 2.0 times higher than those in the controls. The zebrafish exposed to 4 G showed elevated *mc1r* expressions, 1.4 times higher than that in the control. Thus, the PEMF exposure was found to stimulate the expression of melanogenesis-related mRNA.

### 2.3. Western Blotting

Based on the results of the RT-PCR, the expression of the pigmentation-related proteins was measured using Western blot analysis after exposure to 4 G PEMFs at 15 dpf. As shown in Figure 1E, relative to the controls, the expression of all pigmentation-related proteins increased in zebrafish exposed to PEMFs. To investigate the postulated signaling mechanism involved in the effect of PEMFs on pigmentation in zebrafish, zebrafish embryos were treated with PEMFs. In order to study the processes related to pigmentation in the zebrafish exposed to PEMF, we assessed the activation of p-ERK and p-p38 signaling. The protein expression of ERK and p-ERK were determined using Western blotting. Treatment with PEMF led to a significant decrease in phosphorylated-ERK. Conversely, the p-p38 activation was increased in the cells exposed to PEMFs. Thus, the PEMF exposure stimulated melanogenesis in zebrafish.

### 2.4. Pigmentation

**Figure 2:**
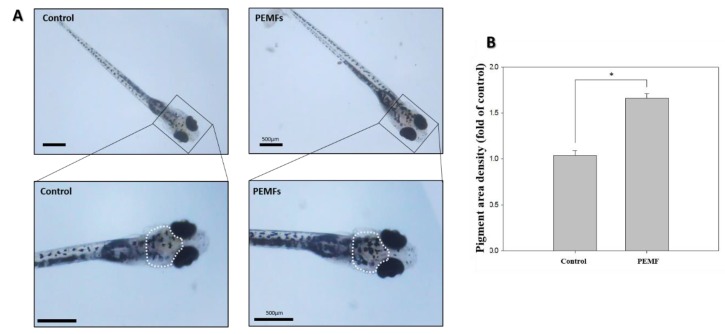
Effects of 4 G PEMF on pigmentation in zebrafish. (**A**) Synchronized embryos (n = 20) were exposed to the PEMF at the indicated intensity and frequency. The effects on zebrafish pigmentation were observed under a stereomicroscope via inferior views of the embryos at 15 dpf. (**B**) The pigmentation area density in the treated embryos, indicated by a white outline, was normalized to that of the control embryos using the ImageJ software. * *p* < 0.05, compared to the control. The scale bar is 500 μm.

A region of interest was selected to measure the pigmentation area, indicated by a white outline embracing the dorsal pigment spot from midway between the eyes, around the pigmented eyes, to the base of the head (Figure 2A). The density of the pigmented area in the treated embryos was normalized to that in the control embryos using ImageJ software. The treatment of the zebrafish embryos with PEMFs for 15 dpf significantly increased the skin melanin formation in the developing larvae (Figure 2B). 

### 2.5. Fontana-Masson Staining

**Figure 3:**
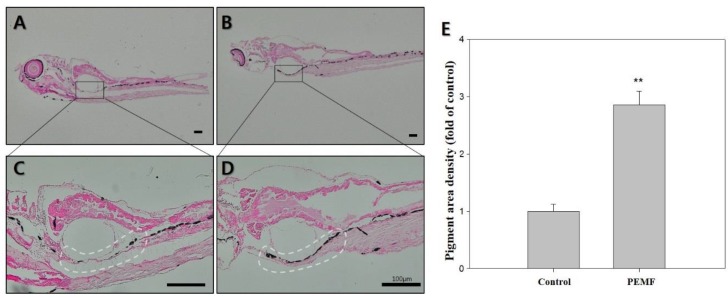
Effects of 4G PEMF in zebrafish at 15 dpf. Representative images of Fontana-Masson-stained zebrafish (dark color indicates secreted melanin). (**A**,**C**) control, (**B**,**D**) EMF. (**E**) the pigmented area density in figure (**A**–**D**) was normalized to that of the control, using ImageJ sotware. ** *p* < 0.05, compared to the control. Original magnification: (**A**,**B**) ×40; (**C**,**D**) × 100 scale bar; 100 μm.

To visualize the melanin, the zebrafish larvae were stained using Fontana-Masson staining, which is used to confirm melanin synthesis. A region of interest was selected to measure the pigmentation area, indicated by a white outline. As shown in Figure 3, compared with the controls, the amount of melanin granules was significantly increased due to PEMF exposure (black color).

## 3. Discussion

A wide variety of approaches have been proposed to increase the synthesis of melanin, including the use of an excimer laser (308 nm) [5,6], UVA1 laser [7], helium–neon laser [8], Nd: YAG laser [9], and Q-switched ruby laser [10,11]. The electric appliances that have magnetic fields in domestic use are easily visible around us, such as microwave cookers (0.0037+/−0.0014 G) and washing machines (0.0027+/−0.0014 G) [27]. On the one hand, electromagnetic fields (EMFs) are abundantly present in modern life, and interest regarding the effect of extremely low frequency (ELF) EMF on human health has been increasing. Different types of magnetic and electromagnetic fields are now used in medical fields, such as in diagnostics (e.g., magnetic resonance, scanning, and microwave imaging) and therapy [28]. Extremely low-frequency electromagnetic fields (ELF-EMF) influence cell differentiation [29,30], cell proliferation, mitochondrial activity [31], cell survival [32], regulate the cell cycle [33], and neurogenesis [15]. Nevertheless, there is a lack of studies examining general EMF effects on melanogenesis. To address this, in a previous study, we evaluated the effect of PEMFs on melanin synthesis in vitro. We found that PEMFs promoted melanogenesis in B16F10 melanoma cells and human melanocyte cells. We used RT-PCR and Western blotting to show that PEMFs stimulate the expression of melanogenesis-related genes and proteins. Thus, our previous findings suggested that the PEMF stimulation significantly promoted melanogenesis [18,19,20]. 

Zebrafish have become a useful model for biochemical studies [24], and have been established as a new in vivo model for evaluating the pigmentation activity of melanogenic regulatory compounds [34]. In the present study, we applied PEMFs for the stimulation of pigmentation in zebrafish. To investigate the hyperpigmentation effect of PEMFs, a melanin assay was used. As shown in Figure 1B, the melanin content increased when the zebrafish were exposed to PEMF. Treatment at 4G strongly increased the melanin content, compared with the control group. Thus, PEMFs were found to promote pigmentation in zebrafish. The pigmentation in the treated embryos was observed under a stereomicroscope at 5 dpf (Figure 2A), and the density of the pigmentation area was normalized to that in the control embryos. The pigmentation area in the untreated zebrafish was clearly less than that in the PEMF treated-zebrafish larvae (Figure 2B). These results are consistent with those reported in our previous in vitro studies [18,19,20].

Pigmentation in zebrafish embryos is due to the activity of some major enzymes, including *tyrp1*, *dct*, and *mitfa*. *Mitf* is the master gene in pigmentation and controls several key mechanisms, such as pigmentation in response to environmental factors, including UV radiation [35]. The activation of MITF induces the expression of the key enzymes of pigmentation, *dct* and *tyrp1*, leading to the production of melanin. In addition, *mc1r* is a key regulator of melanosome dispersal in zebrafish [36].

The expression of these enzymes was determined using RT-PCR, and the results showed that treatment with 4G EMF induced the expression of *mitf* and *dct* (Figure 1D). Moreover, the *dct*, *tyrp1*, and *mitf* expressions, as quantified by Western blotting, increased in the PEMF-treated group. Western blotting using anti-DCT antibody indicated an increase in the expression of these melanophore-specific proteins [37]. Previous studies have demonstrated that MAPK kinases, including ERK and p38, play an important role in pigmentation [38]. It is well established that the ERK signaling pathway is involved in cell proliferation and differentiation; furthermore, the ERK signaling pathway has been identified as a negative regulator of melanogenesis [39]. The upregulation of phosphorylated ERK signaling is related to the reduction of melanin synthesis [20]. However, the phosphorylation of p38 MAPKs can upregulate MITF expression [34,40]. The mitogen-activated protein kinase (MAPK) cascades are an important set of signaling pathways activated in response to EMF in most systems [41]. Thus, to clarify the signaling pathway involved in the PEMF action on melanin synthesis in zebrafish, we examined the phosphorylation of ERK and p38 (Figure 1G,H). The results showed a significant decrease in the phosphorylation of ERK in the PEMF-treated group, which can also lead to the stimulation of the melanogenic pathway by accelerating the MITF activation (Figure 1E,F). The results indicate that the treatment of PEMFs at an optimal intensity and frequency influence the mechanically sensitive kinases, such as ERK and p38. To visualize melanin, the samples were stained with Fontana-Masson staining, and the pigmented area density was determined (Figure 3). Silver nitrate (AgNO_3_) reacts with melanin to produce metallic silver (Ag), resulting in a black stain that can be visualized using a light microscope. As shown in Figure 3, compared with the controls, the amount of melanin granules was significantly increased due to PEMF exposure (dark brown color). The results demonstrate that PEMFs at a specific frequency can stimulate pigmentation in zebrafish. These results may indicate that the optimal condition of PEMFs is good tool for hyperpigmentation therapy for gray hair treatment when melanogenesis is reduced in the hair-follicle, or for hipopigmentation-related skin disorders. As the embryos have been exposed to PEMFs right after birth, more research should be performed to determine whether their effect was a developmental defect.

## 4. Materials and Methods

### 4.1. Zebrafish Maintenance and Embryo Collection

Following standard procedures, adult zebrafish were kept in a circulating filtration system (28 ± 0.5 °C, 14:10 h light:dark) and were fed three times a day. The male and female zebrafish were separated until mating and spawning. After natural spawning, the fertilized embryos were collected and used for the experiments. The embryos were placed individually in each well on 96-well plate filled with 100 µL of water containing sea salt. The embryo and larval developmental stages were expressed in the days post-fertilization (dpf) [42].

### 4.2. PEMFs Exposure

**Figure 4:**
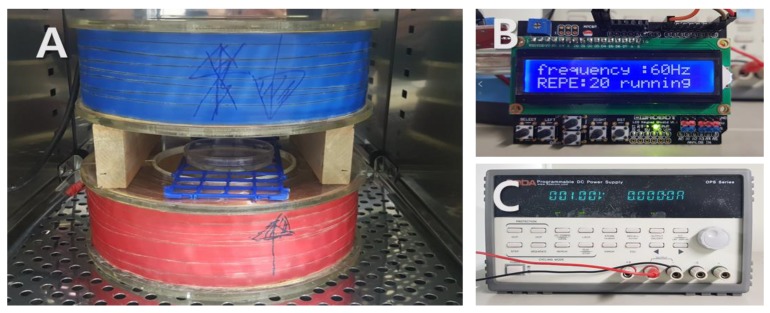
Photograph of the electromagnetic field (EMF) device in incubator. Pulsed EMF was generated using a pair of Helmholtz coils. (**A**) Helmholtz coil; (**B**) function generator; (**C**) power supply.

We used a Helmholtz coil that was able to generate PEMFs; the apparatus is depicted in Figure 4. The stimulus frequency was 60 Hz and teh stimulus wave was in a pulse form. The electromagnetic field device was placed in a 28.5 °C incubator. The unit of measurement of the magnetic density is gauss (G) and the zebrafish embryos were stimulated with PEMFs at intensities of 2, 4, and 20 G for 5 or 15 days. The control group was placed in a separate incubator so as to avoid PEMF exposure. 

### 4.3. Microscopy 

The embryos from the wild-type control group or from the PEMF-exposed groups were observed and photographed under light microscopy so as to examine the pigmentation. 

### 4.4. Melanin Assay

The compound treatment and phenotype-based evaluation were done as previously described [43]. For examining the melanin content, extracts were prepared from 5 dpf zebrafish larvae. Simply put, the embryos were collected and were dissolved in a lysis buffer. After centrifugation, the melanin was extracted into 1 N NaOH, and was incubated at 100 °C for 10 min. The optical density of the supernatant was measured at 405 nm. The results of the treated embryos were compared to those of the appropriate controls, and the final measurements were reported as percentages of the control sample measurements. 

### 4.5. Reverse Transcriptase-PCR Analysis

RT-PCR was used to detect the pigmentation-related genes expressed in the 5 dpf embryos. The total RNA in the zebrafish embryos was extracted using the TRIzol Reagent (Ambion, Austin, TX, USA). The reverse transcriptase reactions were used to synthesize the cDNA from 1 µg of total RNA, using an advantage RT-for-PCR kit (Clontech, Palo Alto, CA, USA). The reverse transcriptase-PCR analysis was performed according to the manufacturer’s instructions. The expression of the genes encoding tyrosinase-related protein 1a (*tyrp1a*), dopachrome tautomerase (*dct* or *tyrp2*), microphthalmia-associated transcription factor a (*Mitfa* or *nacre*), melanocortin 1 receptor (*mc1r*), and gapdh (*glyceraldehyde 3-phosphate dehydrogenase*) were evaluated. The bands were visualized using Molecular Imager ChemiDoc XRS+ (Bio-Rad, Hercules, CA, USA). The software Image J (National Institutes of Health, Bethesda, MD, USA) was used for the quantitative analysis of the results.

### 4.6. Western Blotting

The zebrafish embryos were lysed using a buffer containing 2% SDS, 0.1 mg/mL bromophenol blue in Tris-HCl (pH 6.8), 5% 2-mercaptoethanol, and 10% glycerol, and were boiled at 100 °C for 10 min. The protein was quantified using Bicinchoninic acid (23225, Thermo fisher Scientific, Waltham, MA, USA). Subsequently, the sample was subjected to sodium dodecyl sulphate-polyacrylamide gel electrophoresis, and the protein was transferred from the gel to a nitrocellulose membrane. Anti-DCT (Thermo Fisher Scientific, Waltham, MA, USA), anti-MITF (Origene Technologies, Zug, Switzerland), anti-TRP1 (Abcam, Cambridge, UK), anti-ERK (Cell Signaling Technology, Danvers, MA, USA), anti-p-ERK (Cell Signaling Technology, Danvers, MA, USA), anti-p38 (Antibodies-Online, Aachen, Germany), anti-p-p38 (Thermo Fisher Scientific, Waltham, MA, USA), and anti-β-Actin (Abcam, Cambridge, UK) antibodies were used as the primary antibodies in Western blotting. The blots were incubated with the primary antibodies, and were then further incubated with a horseradish peroxidase-conjugated secondary antibody. The chemiluminescent protein bands were photographed using Molecular Imager ChemiDoc XRS (Bio-Rad, Hercules, CA, USA). To analyze and quantify the Western blotting image, Image J software (National Institutes of Health, Bethesda, MD, USA) was used.

### 4.7. Fontana-Masson Silver Staining

We performed a densitometric analysis to visualize melanin in zebrafish. Fontana-Masson silver staining was performed using a previously described method [44]. Formalin-fixed and paraffin sections were processed on slides and stained with silver nitrate (Kojima Chemical, Kashiwabara, Japan) for 1 h at 56 °C and washed with distilled water. Then, the slides were fixed in 5% sodium thiosulfate solution (Duksan, Seoul, Korea) for 5 min, and were washed with distilled water. After that, they were stained with nuclear fast red solution (Sigma Aldrich, Saint Louis, MO, USA) for 5 min, and were washed with distilled water three times. Finally, after dehydration with 95% ethanol and 100% ethanol, the slides were washed two times with xylene (Duksan, Seoul, Korea).

### 4.8. Statistical Analysis

The data were analyzed using one-way analysis of variance (ANOVA) and Student’s *t*-test. When the value of *p* was <0.05 or <0.01, the difference between the means was considered significant (* *p* < 0.05, ** *p* < 0.01). Graphical representations were produced using the Sigmaplot 2001 software.

## 5. Conclusions

In the present study, we investigated the pro-pigmentation effect of PEMFs in a zebrafish model. Our data suggest that PEMFs promote pigmentation by inducing MITF and DCT, which are mediated through a reduction of ERK phosphorylation and an upregulation of p38 phosphorylation. These results suggest that PEMFs, at an optimal intensity and frequency, are a useful tool for treating gray hair with reduced melanin synthesis in the hair shaft, or hypopigmentation-related skin disorders such as vitiligo.
